# The mediating effect of exhaustion in the relationship between effort‐reward imbalance and turnover intentions: A 4‐year longitudinal study from Sweden

**DOI:** 10.1002/1348-9585.12203

**Published:** 2021-02-05

**Authors:** Constanze Leineweber, Claudia Bernhard‐Oettel, Constanze Eib, Paraskevi Peristera, Jian Li

**Affiliations:** ^1^ Department of Psychology Stockholm University Stockholm Sweden; ^2^ Department of Psychology Uppsala University Uppsala Sweden; ^3^ Department of Environmental Health Sciences Fielding School of Public Health School of Nursing University of California Los Angeles (UCLA) CA USA

**Keywords:** burnout, Sweden, work engagement

## Abstract

**Objectives:**

Earlier studies suggest that imbalance between effort and reward at work associates with exhaustion. Others have found that exhaustion increases turnover intentions; an important precursor of actual turnover that also associates with counterproductive work behaviors. Few, however, have studied the associations between effort‐reward imbalance (ERI) and employees’ intentions to leave their current employment, and whether exhaustion is underpinning that relationship. Here, we investigate the mediating role of exhaustion in the effort‐reward imbalance – turnover intentions relationship.

**Methods:**

Data from three waves covering a time span of four years from the Swedish Longitudinal Occupational Survey of Health (SLOSH) were analysed using structural equation modeling. Cross‐lagged mediation analyses were conducted to estimate if associations from ERI to subsequent turnover intentions were mediated by exhaustion. Other causal directions (direct and reversed direct effects, reversed mediation) were also examined.

**Results:**

A direct path from ERI T1 to turnover intentions T2 was found, but not from ERI T2 to turnover intentions T3. Additionally, results showed that ERI at time points T1/T2 associated significantly with exhaustion two years later (T2/T3). Also, exhaustion at T1 showed a small but statistically significant direct association with turnover intentions at T2 (no association was found between exhaustion T2 and turnover intentions T3). A small, but statistically significant indirect effect from ERI to turnover intentions was found (estimate 0.005; 95% CI 0.002‐0.010).

**Conclusions:**

Providing a good balance between effort and reward for workers is essential to protect employee health and help retain employees in the organization.

## 1 INTRODUCTION

One of the most prominent theoretical models to explain how work environment factors induce strain is the effort‐reward imbalance (ERI) model. This model has its roots in social exchange theories and in the notion of distributive justice.^1^ In short, it assumes that stressful experiences occur if efforts at work are not reciprocated adequately by socially defined rewards (ie, money, esteem and status in terms of promotion prospects and job security).

A large amount of research has shown that higher levels of ERI associate with negative health outcomes, such as cardiovascular diseases,^2^ depression,^3^, ^4^ and lower self‐reported health.^5^ Furthermore, some research studies have investigated how ERI associates with withdrawal behavior (eg lateness, absenteeism, turnover intentions).^5^, ^6^, ^7^, ^8^

From a theoretical point of view, it has been argued that ERI may be linked to withdrawal behaviors because people do not passively remain in a situation where efforts are not adequately rewarded, but instead try to reach a balance, either by maximizing their rewards and/or reducing their efforts.^5^ To reduce efforts, the employee might disengage from the organization either physically through, for example, turnover, absenteeism, or lateness, or psychologically, through for example, lack of creativity or reduced work engagement. Here, we concentrate on one specific form of withdrawal behavior, that is, turnover intentions. Turnover intentions are not only a reliable precursor of actual turnover,^9^ which means considerable expenses for organizations in terms of monetary costs as well as knowledge drainage,^10^ but relate also to other unfavorable outcomes, such as increased counterproductive work behaviors and decreased organizational citizenship behaviors.^11^

Results from previous, mainly cross‐sectional studies,^6^, ^8^, ^12^, ^13^ and some longitudinal studies,^7^, ^14^, ^15^ suggest that high effort, low rewards and their combination, ERI, positively relate to turnover intentions. Many of these studies focus on specific occupational groups, often in human relation occupations, such as nurses, teachers, and health care workers.^7^, ^8^, ^14^, ^15^ For example, one study based on Belgian nurses revealed a positive association between baseline ERI and intentions to leave the current organization as well as the nursing profession one year later.^14^ Similar findings were reported for European nurses, where reward frustration predicted intentions to leave one year later.^7^

Possible underlying mechanisms in the ERI‐turnover intentions relationship have not been studied in much detail. Some researchers have suggested that job satisfaction may be negatively affected by high ERI, which then results in higher turnover intentions.^12^, ^13^ Another underlying mediating factor might be found in burnout/exhaustion. However, evidence regarding the association between ERI and burnout is rather scarce and inconclusive,^16^ although a number of cross‐sectional studies^8^, ^12^, ^17^ and prospective studies^18^, ^19^ suggest a positive association between ERI and burnout. One study found evidence only for reward predicting burnout symptoms two years later.^20^

Finally, there is also some evidence, again predominantly from cross‐sectional studies that emotional exhaustion as a component of burnout relates to turnover intentions.^21^, ^22^, ^23^ Some longitudinal evidence comes from a study on managers who were followed weekly over six weeks: it was found that increases in exhaustion were associated with higher subsequent turnover intentions.^24^ In another study on mental health workers, it was found that increases in emotional exhaustion and decreases in job satisfaction were both significantly associated with increases in turnover intentions six months later.^25^

Putting together evidence from earlier cross‐sectional and prospective studies, it seems plausible to assume that ERI may result in exhaustion and, over time, increase turnover intentions. However, rigorous longitudinal studies investigating these associations in the general working population are lacking. Consequently, our aim of this study is twofold. We investigate (a) whether ERI predicts exhaustion and turnover intentions over time and (b) whether exhaustion mediates the relationship between the ERI and turnover intentions in a large sample of Swedish employees. By doing so, we extend to ERI literature, that has focused much on depression, and sub‐clinical depressive symptoms and less so on exhaustion. Unravelling the underlying mechanisms that link ERI and turnover intentions via exhaustion over time in a large dataset approximately representative for the general working population, this study also provides new insights into how to prevent turnover and thus contributes with knowledge important for employers, unions, and policy makers. By studying reversed effects, that is, effects of turnover intentions on exhaustion and ERI, we also add to the turnover intention literature, which has mainly focused on turnover intentions as an outcome, while ignoring how turnover intentions influence other work‐related behaviors as well as perceptions of the workplace and work environment.

## 2 METHODS

### 2.1 Sample

Data were drawn from the Swedish Longitudinal Occupational Survey of Health (SLOSH) study, which was initiated in 2006 as a follow‐up of participants of the Swedish Environment Survey (SWES) in 2003. Since, data have been collected every second year and participants from later SWES have been added. All participants are invited to answer a paper and pencil questionnaire in two versions, one for those currently in paid work for 30% or more and one for those who work less as they have temporarily or permanently left working life. Today, SLOSH consists of all SWES participants 2003‐2011. For a detailed cohort profile description see Magnusson Hanson et al.^26^

**FIGURE 1:**
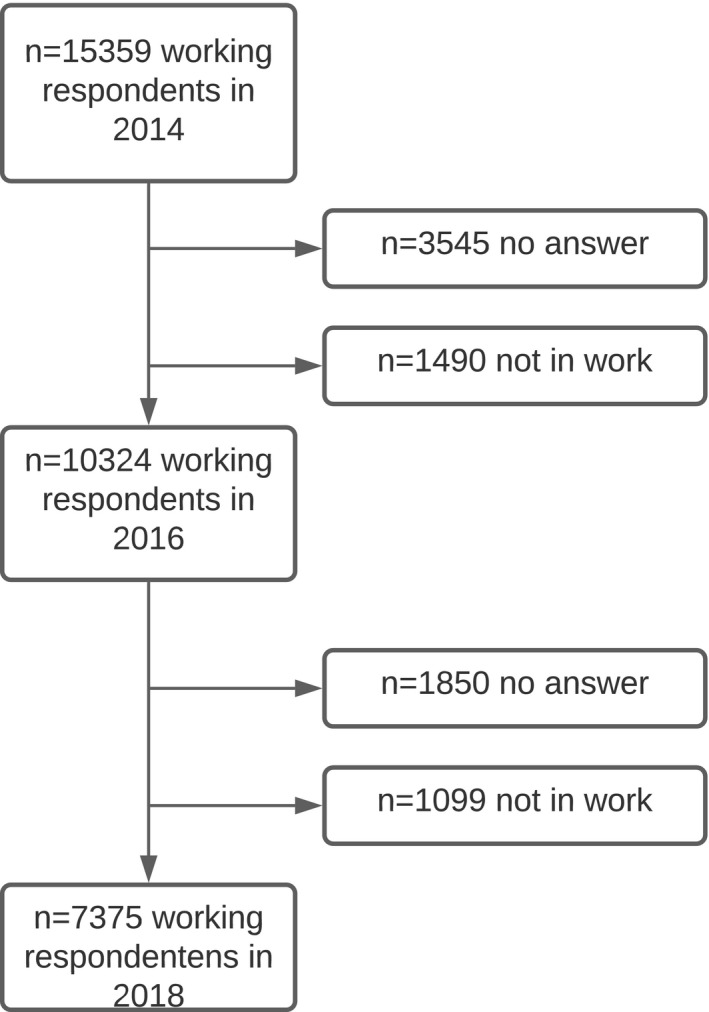
Flow chart to illustrate the sample selection procedure. Participants are drawn from the SLOSH study

The current study is based on participants who answered the questionnaire for those in paid work in the three latest waves of SLOSH, ie 2014 (n = 15 359), 2016 (n = 13 572) and 2018 (n = 11 553). Of the 15 359 participants who answered the questionnaire for those in paid work in 2014, 3545 did not participate in the following wave and a further 1490 answered the questionnaire for those not in paid work. Of the remaining 10 324 participants, in 2018 1850 did not answer the questionnaire and 1099 answered the questionnaire for those not in paid work (for a flow chart see Figure 1). Consequently, the final study sample consisted of 7375 participants (longitudinal response rate = 48%). All participants gave informed consent before taking part.

### 2.2 Effort‐reward imbalance

Effort and reward were measured by the Swedish validated short version of the Effort‐Reward Imbalance questionnaire,^4^ where effort is measured by three items (eg ‘I have constant time pressure due to heavy workload’) and reward by seven items (eg ‘Considering all my efforts and achievements, I receive the respect and prestige I deserve at work’). Reward consists of three components: ‘esteem’ and ‘job security’, each represented by two items, and ‘job promotion’, represented by three items. Items are answered on a four‐point Likert‐scale ranging from 1 = agree totally to 4 = do not agree at all. Where needed, items were reversed. The sum score for each of the two dimensions, effort and reward, was calculated, such that higher scores reflect higher effort and higher rewards. The Cronbach's alpha for effort was 0.78 for all three waves and varied between 0.71 and 0.72 for reward. Finally, an effort‐reward ratio was constructed. This was done based on a predefined algorithm (effort score divided by reward score, multiplied by the ratio of the number of items). Higher values indicate higher imbalance.

### 2.3 Exhaustion

Exhaustion was measured by six of eight items of the Shirom‐Melamed Burnout Measure (SMBM),^27^, ^28^ namely ‘I feel tired’, ‘I feel “fed‐up”’, ‘My “batteries” are “dead”’, ‘I feel burned out’, ‘I feel mentally fatigued’, and ‘I feel no energy for going to work in the morning’. All items are answered on a 7‐point scale ranging from 1 (‘almost never’) to 7 (‘almost always’). Two items of the original scale, ie ‘feeling refreshed’ (the only positively formulated item) and ‘I feel physically exhausted’ (the only item relating to physical fatigue), provided relatively low factor loadings and rendered that scale no time invariant and were consequently excluded. In excluding the positive formulated item, we follow recommendations from Shirom & Melamed.^28^ To measure exhaustion, a mean score of the remaining 6‐item scale was calculated. This measure showed high internal consistency (Cronbach's alpha varied between 0.90 and 0.91) and was found to be time invariant (scalar invariant), indicating that relations between the items and the latent factor were similar and means were comparable across time.

### 2.4 Turnover intentions

Intentions to leave one's job were measured by a single question ‘I feel like resigning from my current employment’ with five response options ranging from ‘strongly disagree’ (1) to ‘strongly agree’ (5).^29^

### 2.5 Covariates

We controlled for age, gender, and income, which were all obtained by linkage of questionnaire data to registries by means of the unique Swedish ten‐digit personal identification number. Age is age at baseline year (2014), gender was coded into ‘0’ for male and ‘1’ for female, and income is gross income during the year in Swedish crowns. Finally, we control for change of job, because some people may have changed job between waves, which could influence their ERI, exhaustion and turnover intentions. Information on job turnover was obtained from questionnaire data (‘The following questions apply to your place(s) of work in the past two years: Have you changed work?’) and coded into ‘yes’ and ‘no’.

### 2.6 Analytical strategy

To test the hypotheses, we specified and tested various structural models. We used cross‐lagged panel models for mediation analyses, which take stability and correlation of measurements over time into account.^30^ Maximum likelihood estimates based on the MLR estimator are reported and full information maximum likelihood (FIML) was utilized to reduce bias due to missing data.^31^ In order to set the independent and dependent variables on one metric and account for measurement error in single indicator variables, the observed variables ERI and turnover intentions were translated into latent variables with a single indicator where the residual variances were fixed to (1‐ reliability) * sample variance.^32^ We assumed a reliability of 0.70.^33^ In the modeling process, for a more parsimonious model lagged effects were not constrained to be equal. Specifically, we tested the following structural models (see Appendices, Figure S1):

(a) An *autoregressive model* (Model 1) included the temporal stability effects between the constructs. For instance, ERI at Time 1 (T1) was specified to predict ERI at Time 2 (T2), and ERI at T2 was set to predict ERI at Time 3 (T3). At every time point, all variables were set to correlate freely. (b) A *direct effects model* (Model 2) extended the baseline model by adding the cross‐lagged effect from ERI to exhaustion and from ERI to turnover intentions. That is, ERI at T1 was set to predict exhaustion and turnover intentions at T2 and ERI at T2 was set to predict exhaustion and turnover intentions at T3. (c) A *mediation model* (Model 3) extended Model 2 by adding paths from exhaustion to turnover intentions. (d) A *reversed direct effects model* (Model 4) extended Model 3 by including the reversed cross‐lagged effects. Specifically, exhaustion at T1 (T2) was set to predict ERI at T2 (T3). (e) A *full effects model* (Model 5) extended Model 4 by including additional reversed cross‐lagged effects from turnover intentions at T1 (T2) to exhaustion T2 (T3) and ERI at T2 (T3). In all models, we controlled for age, gender, and income at Time 1, and for change of job at Time 2 and Time 3. The significance and confidence intervals for the total indirect effect were assessed with bootstrap estimation (10 000 samples).

Differences in model fit were assessed based on the chi‐square difference test for continuous non‐normal variables for nested models.^34^ Model fit was assessed with the comparative fit index (CFI), the root mean square error of approximation (RMSEA) and the standardized root mean square error of approximation (SRMR). RMSEA values lower than 0.06, CFI and TLI values above 0.90 and SRMR values smaller than 0.10 were considered to indicate acceptable model fit.^35^, ^36^ Sample statistics were analyzed in SAS 9.4, SEM analyses were conducted in Mplus (version 8).

## 3 RESULTS

### 3.1 Descriptive characteristics

Sample characteristics are found in Table 1. The mean age of the sample was about 50 (SD = 8.69) years at baseline. Around 60% of the sample were women and the mean yearly income was 392 thousand Swedish crowns (SD = 182.52). Around 20% changed job between T 1 and T2 and between T2 and T3 respectively.

**TABLE 1:** Descriptive characteristics of the sample (n = 7375)

	2014	2016	2018
Age, yrs (mean ± SD)	49.94 ± 8.69	51.94 ± 8.69	53.94 ± 8.69
Women, % (n)	58.79 (4336)	58.79 (4336)	58.79 (4336)
Yearly income, in thousand SEK (mean ± SD)	392 ± 182	423 ± 196	453 ± 208
Job turnover during the past two years, % (n)	—	20.75 (1520)	20.95 (1536)
Effort‐reward imbalance, range 0.2‐4.0 (mean ± SD)	1.08 ± 0.43	1.07 ± 0.41	1.05 ± 0.41
Exhaustion, range 1‐7 (mean ± SD)	2.46 ± 1.17	2.48 ± 1.22	2.48 ± 1.24
Turnover intentions, range 1‐5 (mean ± SD)	1.75 ± 1.14	1.81 ± 1.17	1.84 ± 1.21

### 3.2 Model testing

**FIGURE 2:**
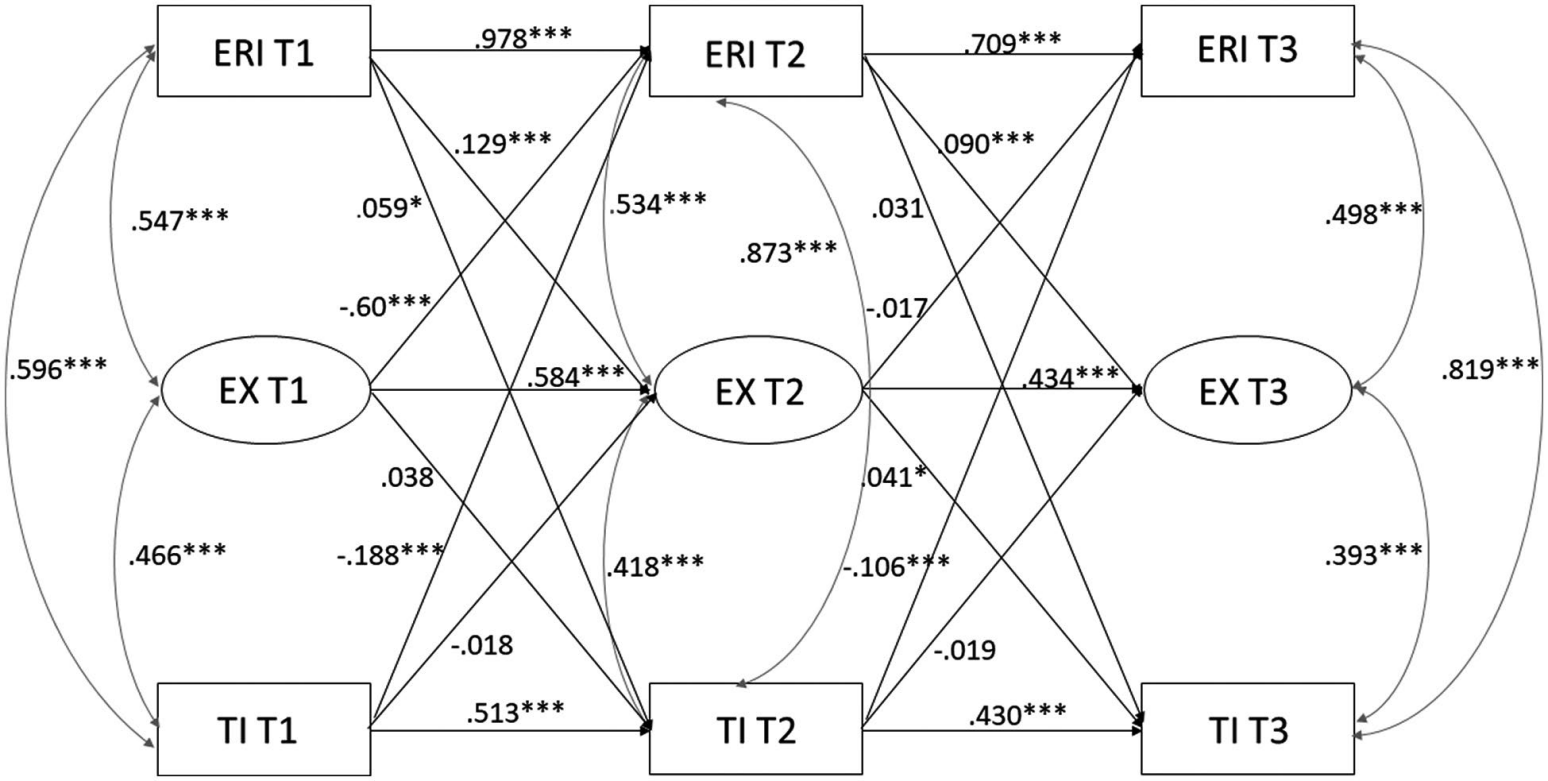
Structural equation model parameters and *P*‐values for paths among effort‐reward imbalance, exhaustion and turnover intentions (Model 5). Note: Standardized estimates provided (except for covariates and autocorrelations between T1 and T3); *P ≤ .05; ***P ≤ .001

Model fit for the different models are presented in Table 2. The full model (Model 5) was the best fitting model (see Figure 2). This model showed significant direct paths from ERI at one time point to exhaustion (EX) two years later (ERI T1→EX T2 *β* = 0.129, *P* = .000; ERI T2→EX T3 *β* = 0.090, *P* = .000). ERI at T1 showed also an association with turnover intentions at T2 (*β* = 0.059, *P* = .039), but this association became non‐significant from ERI T2 to turnover intentions T3 (*β* = 0.031, *P* = .192). The model also revealed a borderline significantly direct path from exhaustion T1 to turnover intentions T2 (*β* = 0.038, *P* = .051), which reached statistical significance from T2 to T3 (*β* = 0.041, *P* = .030). The indirect effect estimate over all three time points was 0.005 (*bootstrap* 95% CI 0.001 to 0.010); that is, for every 1‐unit increase of the mean score of ERI (ranging from 0.2 to 4.0) an increase (ranging from 0‐1) of 0.005 in turnover intentions goes via exhaustion. In addition to the causal direct paths and the indirect path, also a number of reversed paths were significant, namely a direct path from exhaustion at T1 to ERI at T2 (*β* = −0.060, *P *= .005), but not from exhaustion T2 to ERI T3 (*β* = −0.017, *P *= .318). Furthermore, direct paths from turnover intentions at one time point to ERI two years later were revealed (TI T1→ERI T2 *β* = −0.188, *P *< .001; TI T2→ERI T3 *β* = −0.106, *P *< .001). No direct paths from turnover intentions to exhaustion at the next time point were found (TI T1→EX T2 *β* = −0.018, *P* = .366; TI T2→EX T3 *β* = −0.019, *P *= .276).

**TABLE 2:** Model fit table for effort‐reward imbalance, burnout, and turnover intentions, controlled for age, gender, income, and job change

	Chi^2^	*df*	Chi^2^ diff^a^	RMSEA	CFI	TLI	SRMR
Model 1: Stability effects	7795.588	362		0.053	0.926	0.917	0.055
Model 2: M1 + ERI →EX, TI	7728.174	358	vs. Model 1: *P *< .0001	0.053	0.926	0.916	0.054
Model 3: M2 + EX→TI	7707.557	356	vs. Model 2: *P *< .0001	0.053	0.926	0.916	0.054
Model 4: M3 + EX→ERI	7685.927	354	vs. Model 3: *P *< .0001	0.053	0.927	0.916	0.053
Model 5: M4 + TI→ERI, EX	7600.779	350	vs. Model 4: *P *< .0001	0.053	0.927	0.916	0.053

Note

N = 7287.

Abbreviations: CFI, comparative fit index; *df*, degrees of freedom; ERI, effort‐reward imbalance; EX, exhaustion; RMSEA, root‐mean‐square error of approximation; SRMR, standardized root‐mean‐square residual; TI, turnover intentions.

^a^ Satorra‐Bentler Scaled Chi‐Square difference test

Among covariates, age associated negatively with ERI (*r* = −.056, *P *< .001), exhaustion (*r* = −.076, *P *= .001), and turnover intentions (*r* = −.168, *P *= .001). Being female was positively associated with ERI (*r* = .120, *P *= .001) and exhaustion (*r* = .100, *P *= .001), but not turnover intentions (*r* = .017, *P *= .206). Income associated negatively with ERI (*r* = −.054, *P *= .001) and exhaustion (*r* = −0.059, *P *= .001) and positively with turnover intentions (*r* = .037, *P *= .006). Having changed the job during the previous two years associated negatively with ERI both at T2 (*r* = −.232, *P *= .001) and T3 (*r* = −.265, *P *= .001), exhaustion both at T2 (*r* = −.050, *P* = .001) and T3 (*r* = −.091, *P *= .001), and turnover intentions both at T2 (*r* = −.174, *P *= .001) and T3 (*r* = −.173, *P *= .001).

## 4 DISCUSSION

This paper had two overall aims: Firstly, we investigated ERI as a possible predictor of exhaustion and turnover intentions over time. Secondly, we studied if exhaustion would function as a mediator in the ERI‐turnover intentions relationship. To further explore relationships between ERI, exhaustion and turnover intentions over time, we additionally tested for reversed mediating and reversed direct paths, and adjusted for a number of potential confounders as control variables.

Our results confirm a strong cross‐sectional association between ERI and turnover intentions in the way that experiencing poor balance between effort and reward associates with turnover intentions measured at the same point in time. However, as such associations allow no conclusions to be drawn about the direction of the association, our main interest was to investigate if ERI *precedes* turnover intentions. However, we found only inconsistent support for ERI being a precursor of later turnover intentions; ERI at T1 predicted turnover intentions at T2, but ERI at T2 did not predict turnover intentions at T3. One possible reason might be that turnover intentions were on average rather low in our sample (about 1.8 on a scale from 1 to 5, see Table 1). Also, as we included auto‐regressions as well as cross‐sectional relationships little variance was left to be explained.

Turning our attention to the association between ERI and exhaustion, we can see that ERI is strongly associated with subsequent exhaustion, also when taking cross‐sectional correlations into account. Thus, our finding resembles previous cross‐sectional^8^, ^12^, ^17^ and prospective studies^18^, ^19^ that report positive associations between ERI and burnout. Furthermore, we found that exhaustion predicted subsequent turnover intentions. Our finding thus supports earlier finding that suggest that burnout is a predictor of turnover intentions.^24^, ^25^, ^37^ This finding has earlier been explained in the way that withdrawal behaviors such as turnover intentions may be used in order to lower or eliminate work stressors and are thus used as an attitudinal coping strategy when burnout reduces the required individual resources within the work context.^38^

Taken together, our results indicate that exhaustion mediates the relationship between ERI and turnover intentions. Even though the indirect effect estimate was small in size, this needs to be put in relation to the fact that it was tested with a rather strict test considering all possible cross‐sectional and reverse causation paths. Also, as suggested by previous research, there are other mediating mechanisms apart from emotional exhaustion^12^, ^13^ and future research may need to test what emotional exhaustion means relative to other such mediation pathways to better understand the ERI‐turnover intentions relationship.

When studying the ERI‐turnover intentions relationship, we also controlled for other factors relating to turnover intentions. Two important contributing factors to ERI seem to be income and prior actual turnover. While one might expect that those with lower income would report higher turnover intentions, we found that those with higher income actually reported higher turnover intentions. At the same time, income was positively associated with lower levels of ERI and exhaustion. A possible explanation might be that those with higher income have also other resources to dispose of, for example, in terms of education and work experience, which makes them more attractive in the labor market. Also, those who had changed workplace during the two preceding years reported significantly lower levels of ERI, exhaustion, and turnover intentions. This is indirectly suggested by Siegrist,^39^ who proposed that a lack of reciprocity in terms of high cost and low gain occurs frequently where workers have no alternative choice in the labor market, implying that those who have alternatives will change their situation. This idea is also supported by a previous study that showed that both high ERI and available employment opportunities were independently associated with intentions to leave the nursing profession.^15^

In addition to the causal path from ERI to turnover intentions and exhaustion, we also found some reversed causal paths. For one, exhaustion was negatively associated with ERI, such that higher levels of exhaustion predicted lower levels of ERI two years later, though the association reached only statistical significance between T1 and T2. Second, turnover intentions associated negatively with ERI, indicating that higher levels of intentions to leave were associated with lower levels of ERI two years later. These findings seem at the first glance surprising. One possible explanation is that people who experience high levels of exhaustion and/or turnover intentions at one point in time, do successfully cope with these feelings, for example, in terms of finding a new job, job crafting,^40^ or acceptance,^41^ and thus report lower ERI at the next time point.

When inspecting our findings, it is of importance to keep in mind that measurement points were two years apart. This time span might have been better suited to uncover associations with reactions that need some time to develop. While turnover intentions might be a rather spontaneous reaction to events in the work environment, feeling of exhaustion may take longer time to develop. Indeed, Zapf et al^42^ suggest specific time relationships of stressors and strains, where time lags that are too long leads to an underestimation of the true causal impact. Consequently, our study design might not have been optimal to uncover potential longitudinal effects from ERI to turnover intentions, but better suited to capture relations between ERI and exhaustion. However, most earlier studies on ERI and its consequences used time lags of one or two years^7^, ^14^, ^18^, ^20^ and until now, there is little guidance for researchers to plan for optimal time lags in longitudinal studies.^43^ To find an optimal time lag may be particularly problematic if the intention, as in our study, is to include a large population, and to measure both health‐related and behavioral intention effects that may need different time lags to unfold. Our time span of two years between measurements may also explain the reversed effects. Most probably it will take some time to effectively cope with turnover intentions eg by job crafting, which would be mirrored in the negative effect from turnover intentions at one point in time to ERI two years later. Further studies with repeated measures with different time lags are needed to shed light on the timely development among ERI, exhaustion, and turnover intentions.

### 4.1 Strength and limitations

Strengths of our study are the prospective design with three repeated waves of measurement and the use of a statistical method that accounted for stability and correlation of measurements over time of all variables. The use of a study population approximately representative of the working population allows for a better generalizability as many of the earlier studies investigating ERI in relation to exhaustion and turnover intentions that focus on specific professions, often in the health care sector. Still, as our study population had a mean age of 50 and reported low levels of turnover intentions, results might not be generalizable to younger populations or populations who generally report more turnover intentions. Using only one of the sub‐dimensions of burnout, we were not able to investigate associations between ERI and other sub‐dimensions such as cognitive weariness. Also, sample attrition might have affected our results, where especially those with the highest levels of exhaustion might be more likely to not participate in the study and/or to leave employment (and thus are not included in the study sample). While we controlled for some of the most important possible confounders, reasons for experiencing turnover intentions are manifold and there might be other factors from the private life, such as family issues, or from the psychosocial work environment, such as social aspects at the workplace or emotional demands, that might influence turnover intentions, but which we could not control for.

## 5 CONCLUDING REMARKS

ERI has previously been shown to associate negatively with a number of health outcomes. Here, we could show that ERI not only relates to exhaustion, but also to turnover intentions. Turnover intentions are an important predictor of actual turnover, but are also related with lowered discretionary behaviors at work^11^; two phenomena related with considerable costs for organizations. Consequently, organizations should strive to reduce turnover intentions among their employees. Our results show that one way to do so is to improve employees’ effort‐reward balance. There are several ways organizations can improve the effort‐reward ratio for their employees. On the organizational level, ERI can be improved by reducing efforts and work demands while simultaneously improving rewards, this can, for example, be done as part of the regular monitoring of work environment factors and the incentives provided at the workplace. On the individual level, a dialogue between manager and employee on mutual expectations regarding efforts and rewards may be important as part of the communication and managing of the psychological contract.^44^ Also, stress management interventions at work has been shown to be particularly effective for improving the effort‐reward ratio.^45^ Providing employees with psychotherapeutic interventions or coaching to reduce exhaustion and improve stress management may perhaps also help tackle aspects of burnout and turnover intentions. Importantly, any intervention may be most promising if it addresses both the individual and the organizational level.^46^

## DISCLOSURE

Ethical permission for SLOSH (#2017/2535‐32) and the current study (#2018/1439‐32) were obtained by the Regional Research Ethics Board in Stockholm. All study participants gave their informed consent. Authors declare no Conflict of Interests for this article.

## AUTHOR CONTRIBUTIONS

CL, C.B‐O., and CE conceived the ideas; CL analyzed the data; all authors interpreted the data for the work; CL wrote the first and subsequent drafts of the work; all authors revised it critically for important intellectual content; all authors approved the final version to be published.

## Supporting information

Figure S1
