# Supplementary figures and images for: The mediating effect of exhaustion in the relationship between effort‐reward imbalance and turnover intentions: A 4‐year longitudinal study from Sweden
Source: J Occup Health. 2021 Feb 5;63(1):e12203. doi: 10.1002/1348-9585.12203 (PMC7862986; doi:10.1002/1348-9585.12203)

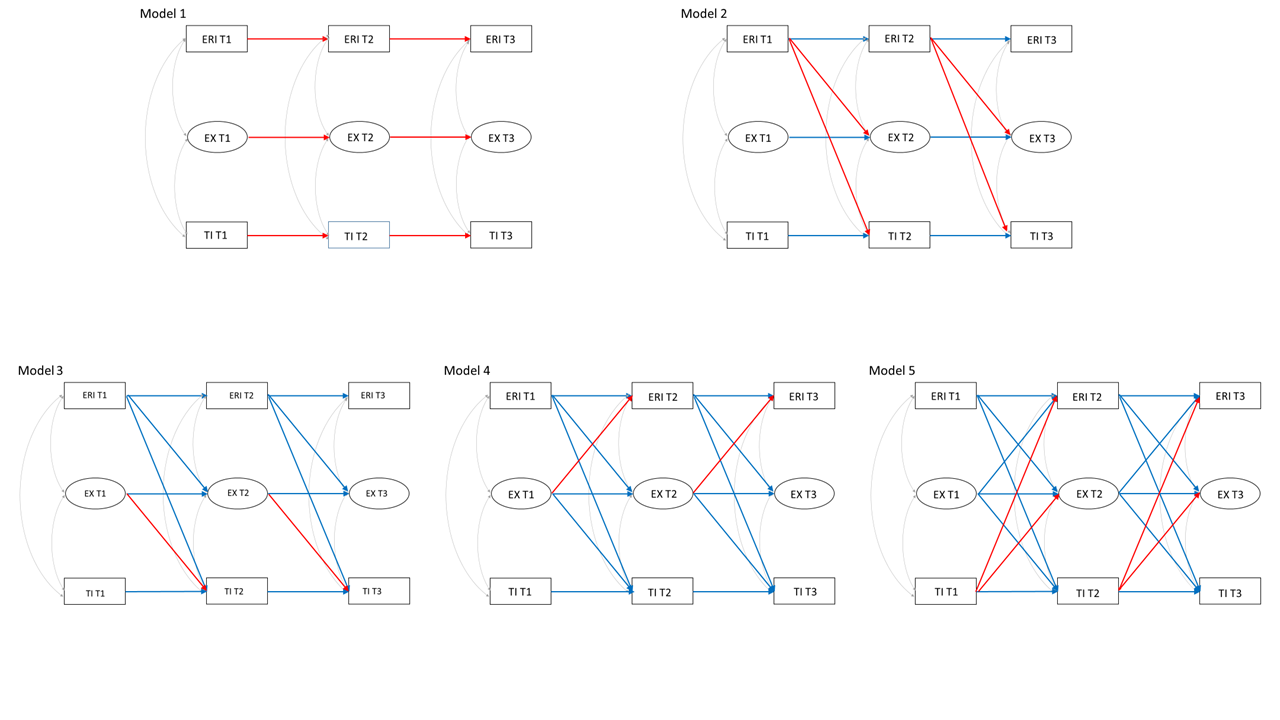

Supplement: Supplementary file 1 — Figure S1 [file JOH2-63-e12203-s001.tif]
